# Renal Cell Carcinoma Texture Analysis With the KiTS23 Dataset: A Retrospective Study

**DOI:** 10.1155/aiu/5082571

**Published:** 2026-08-19

**Authors:** Yuan Liang, Abraham G. Campbell, Sourav Bhattacharjee

**Affiliations:** ^1^ School of Computer Science, University College Dublin, Dublin, Ireland, ucd.ie; ^2^ School of Veterinary Medicine, University College Dublin, Belfield, Dublin, Ireland, ucd.ie; ^3^ Conway Institute of Biomolecular and Biomedical Research, University College Dublin, Belfield, Dublin, Ireland, ucd.ie; ^4^ UCD One Health Centre, University College Dublin, Belfield, Dublin, Ireland, ucd.ie; ^5^ UCD Earth Institute, University College Dublin, Belfield, Dublin, Ireland, ucd.ie; ^6^ Institute for Functional Anatomy, Charité–Universitätsmedizin Berlin, Campus Charité Mitte Philippstraße 11, Berlin 10115, Germany, charite.de

**Keywords:** computed tomography texture analysis, feature selection, logistic regression, machine learning, radiomics, renal cell carcinoma

## Abstract

**Background and Aims:**

Computed tomography texture analysis, powered by machine learning techniques, may differentiate clear cell renal cell carcinoma (ccRCC) from other renal tumor subtypes, such as papillary and chromophobe variants, or benign renal masses such as oncocytomas, as demonstrated here using the KiTS23 dataset.

**Methods:**

After excluding multifocal cases to avoid lesion‐level labeling ambiguity and intrapatient lesion heterogeneity, 396 cases were included. Using PyRadiomics, 386 radiomics features were initially extracted from preprocessed computed tomography volumes and tumor segmentation masks. Subsequent multistage feature selection reduced the candidate feature set to 73 radiomics descriptors, and LASSO further selected six features for the primary classifier. Candidate machine‐learning models were evaluated, with class‐weighted Logistic Regression selected as the primary model. Threshold adjustment using the Youden index and bootstrap feature‐selection stability analysis were performed.

**Results:**

The LASSO‐selected class‐weighted Logistic Regression model showed the best overall performance. Using the default probability threshold of 0.5, it achieved an accuracy of 76.5%, an area under the receiver operating characteristic curve of 80.4%, a precision of 91.2%, a sensitivity of 73.8%, and a specificity of 82.9% on the held‐out test set. Youden‐index thresholding increased sensitivity but reduced specificity; therefore, the fixed threshold of 0.5 was retained as the primary operating threshold. Bootstrap stability analysis showed that several LASSO‐selected radiomics descriptors were repeatedly selected across resampled training cohorts.

**Conclusions:**

Radiomics‐based machine learning shows promise for discriminating ccRCC from non‐ccRCC renal tumors, but the results remain preliminary and have been evaluated internally. Given the moderate discrimination and lack of external validation, further studies using larger, multicenter datasets with standardized radiomics workflows are necessary.

## 1. Introduction

**FIGURE 1:**
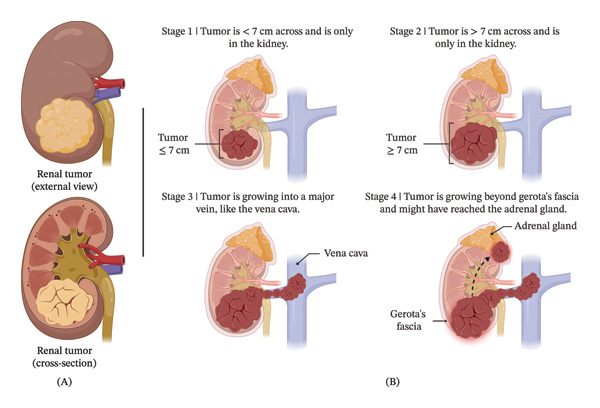
The kidney tumor. (A) A kidney tumor is shown as a renal mass from both external and renal cross‐sectional views. (B) The staging of renal tumors (stages 1–4). Gerota’s fascia is a thin fibrous (collagen) fascia that wraps around the kidneys and adrenal glands. In addition to the kidneys and adrenal glands, it contains the perinephric fat layer. Figure created using Biorender.com.

According to the Global Cancer Observatory, there were an estimated 431,288 new cases of renal cancer in 2020 [1, 2]. Renal cell carcinoma (RCC), an adenocarcinoma predominantly arising from cells lining the proximal convoluted tubules, is the commonest renal tumor and accounts for 90%–95% of all renal cancers [3–6]. It has a male‐to‐female ratio of 2:1 and is commonly detected in elderly patients (> 75 years). Common symptoms include hematuria (40%), flank pain (40%), emaciation (33%), and a feeling of a mass in the abdomen/flank (25%), followed by fever and hypertension (20%) [7, 8]. The 5‐year survival rate for localized lesions is 65%–90%, although it may be considerably reduced (25%–30%) once metastasis sets in, with spread commonly noted in the lungs, liver, and brain (Figure 1) [9, 10].

Increased sensitivity of advanced diagnostic techniques, such as computed tomography (CT) [11, 12], has led to a significant rise in RCC incidence. CT is widely accepted as the investigation of choice for the detection, characterization, and staging of RCC. Within the RCC cohort, clear cell RCC (ccRCC) constitutes the majority (75%), leading to the predominant number of fatalities [13]. Previous research has shown that CT texture analysis (CTTA) can be used to predict malignancy subtyping, malignant versus benign discrimination, and nuclear grading [14–17]. This study evaluated the effectiveness of CTTA in differentiating ccRCC from non‐ccRCC renal tumors, including papillary RCC, chromophobe RCC, and renal masses that may be confused with RCCs, such as oncocytoma.

However, systematic reviews note significant methodological variability [14] and call for standardized multicenter data. This study addressed this gap by applying a structured radiomics workflow to the publicly available KiTS23 dataset, focusing on the classification of ccRCC against other subtypes. Hence, in this study, classical machine‐learning classifiers were selected for their interpretability and established use in radiomics, while deep‐learning approaches remain available for future studies involving larger, multicenter datasets.

## 2. Materials and Methods

### 2.1. KiTS23 Dataset

**FIGURE 2:**
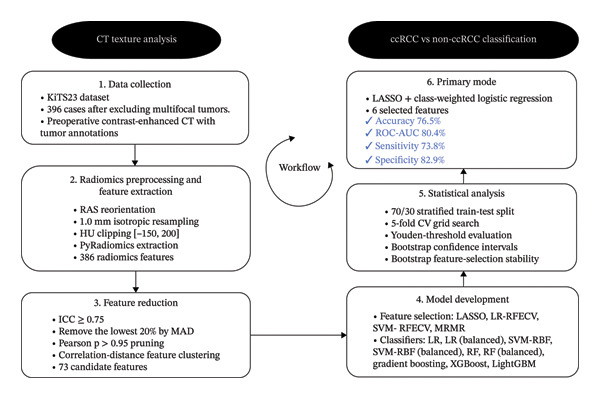
Workflow of the CT texture analysis pipeline, including cohort selection, radiomics preprocessing and feature extraction, feature reduction, model development, statistical evaluation, and primary classifier performance.

The KiTS23 dataset is part of the Kidney Tumor Segmentation Challenge 2023 (KiTS23, https://kits-challenge.org/kits23/), an extension of its previous iterations, including KiTS19 and KiTS21, aimed at advancing the performance of machine‐learning models for kidney tumor segmentation in medical images [18]. The dataset is specifically designed to aid in the automatic segmentation of kidney tumors and surrounding structures from CT scans, supporting the development of tools for more accurate and efficient diagnosis and treatment planning for renal cancer. The KiTS23 training set was used as the source cohort for this study. After excluding multifocal cases to avoid lesion‐level label ambiguity and intrapatient lesion heterogeneity, the final cohort used for radiomics modeling comprised 396 cases (Figure 2).

The dataset consists of preoperative contrast‐enhanced abdominal CT scans from patients who underwent partial or radical nephrectomy for renal tumors. It includes imaging data from a diverse range of clinical settings, capturing a wide variety of tumor sizes, shapes, and stages, as well as various kidney and anatomical variations. Each image is accompanied by ground‐truth annotations that are meticulously curated to facilitate robust training and evaluation of segmentation algorithms.

Although the KiTS23 dataset includes cases from different centers and varying CT acquisition protocols, which introduces some heterogeneity, the lack of external validation remains a limitation. Future studies should include independent datasets, such as TCGA‐KIRC, to further evaluate the model’s generalizability.

### 2.2. Ethics Approval and Consent Statement

The KiTS23 dataset was originally approved by the University of Minnesota Institutional Review Board (study #1611M00821) for data collection and sharing in de‐identified form. This secondary analysis of anonymized public data did not require additional ethics approval. Written informed consent was obtained from all participants by the original investigators.

### 2.3. Feature Extraction

Radiomics features are quantitative descriptors extracted from medical images, such as CT scans, that capture information about the texture, shape, intensity, and spatial heterogeneity of tissues, lesions, or tumors. Regarding the KiTS23 dataset, radiomics features were derived from preoperative CT scans and tumor segmentation masks to characterize kidney tumors (Figure 2).

Before feature extraction, all CT volumes and segmentation masks were reoriented to a common RAS axis convention and resampled to an isotropic voxel spacing of 1.0 mm. Bilinear interpolation was used for CT images, while nearest‐neighbor interpolation was used for segmentation masks to preserve label integrity. CT intensities were clipped to a fixed Hounsfield Unit (HU) range of [−150, 200] before downstream processing. No image‐level z‐score normalization was applied before radiomics extraction, given that such normalization may alter first‐order intensity descriptors. When required for machine‐learning models, feature standardization was applied only after radiomics extraction.

Radiomics descriptors were extracted from the preprocessed CT volumes using PyRadiomics [19]. Feature computation followed IBSI‐aligned settings to improve reproducibility [20]. Shape features were computed from the original image type only, whereas first‐order and texture features were computed from both the original image and Laplacian‐of‐Gaussian filtered images with *σ* ∈ {1, 2, 3}. Intensity discretization was performed using a fixed bin width of 25 HU during radiomics extraction. This procedure yielded an initial radiomics feature vector of 386 features for each case.

The extracted features included first‐order intensity statistics, 3D shape descriptors, and texture features derived from GLCM, GLSZM, GLRLM, GLDM, and NGTDM matrices. These feature classes were used to characterize tumor intensity distribution, morphology, and intratumoral heterogeneity for subsequent machine‐learning analysis.

### 2.4. Feature Selection

Feature selection was performed to reduce dimensionality, minimize redundancy, and improve model robustness. Following radiomics extraction, the initial feature vector comprised 386 features. A multistage feature‐reduction strategy was first applied before model construction. Features with interobserver reliability (ICC) < 0.75 were removed [21]. ICC was assessed using the subset of single‐lesion KiTS23 cases with three independent tumor annotations. Among the 396 single‐lesion cases included in the modeling cohort, 280 cases had tumor masks from all three annotators and were used for ICC analysis, whereas the remaining cases had only one available annotation and were therefore not suitable for ICC calculation. For each radiomic feature, values extracted from the three annotators’ masks were arranged in a cases‐by‐observers matrix, and ICC (3, 1), a two‐way mixed‐effects consistency single‐measure model, was calculated. Features with ICC < 0.75 were excluded from subsequent modeling. The lowest 20% of features ranked by median absolute deviation were then excluded to remove low‐variability descriptors. Highly correlated feature pairs were pruned using a Pearson correlation threshold of |*ρ*| > 0.95. Finally, feature clustering based on correlation distance was applied using a distance threshold of 0.2, and a representative feature was retained from each cluster. This procedure yielded a 73‐feature candidate radiomics set.

Model‐specific feature selection was subsequently performed using four candidate strategies: least absolute shrinkage and selection operator (LASSO) [22], Logistic Regression‐based recursive feature elimination with cross‐validation (LR‐RFECV), linear support vector machine‐based recursive feature elimination with cross‐validation (SVM‐RFECV), and minimum redundancy maximum relevance (mRMR) [23]. These methods were evaluated to examine whether different feature‐selection strategies produced consistent classifier performance. The final primary model used six LASSO‐selected features in a class‐weighted Logistic Regression classifier to balance discrimination performance, specificity, and interpretability. Bootstrap feature‐selection stability analysis was also performed to assess how frequently individual features were retained across resampled training cohorts.

### 2.5. Statistical Analysis

The objective of the statistical analysis was to distinguish ccRCC from non‐ccRCC renal tumors, including papillary RCC, chromophobe RCC, and oncocytoma. After exclusion of multifocal cases, the final cohort comprised 396 cases. A stratified case‐level train‐test split was used, with 70% of cases assigned to the training set and 30% assigned to the held‐out test set. Model selection and hyperparameter tuning were performed within the training set, and final performance was evaluated on the held‐out test set.

Statistical analyses and reporting were conducted in accordance with the recommendations outlined previously in the “Guidelines for reporting of statistics for clinical research in urology” and the SAMPL guidelines [24, 25]. As this investigation represented a retrospective radiomics‐based diagnostic modeling study, reporting was guided by the TRIPOD statement to improve transparency and reproducibility [26]. Radiomics feature extraction and reporting was aligned with IBSI recommendations to improve reproducibility [20].

i. Model training
 The 73‐feature candidate radiomics set was used as input for model‐specific feature selection and classifier training. Candidate classifiers included Logistic Regression, class‐weighted Logistic Regression, radial‐basis‐function SVM (SVM‐RBF), class‐weighted SVM‐RBF, Random Forest, class‐weighted Random Forest, Gradient Boosting, XGBoost, and LightGBM. Hyperparameters were optimized using a five‐fold cross‐validated grid search in the training set, with ROC‐AUC used as the optimization metric.
 Class‐weighted models were explicitly evaluated in the analysis. In addition to the default probability threshold of 0.5, Youden‐index threshold optimization was performed using cross‐validated out‐of‐fold predictions from the training set. The selected Youden threshold was then applied unchanged to the held‐out test set. The primary model was selected according to overall held‐out test performance, specificity, and interpretability.
ii. Quantitative evaluation
 Model performance was evaluated using accuracy, ROC‐AUC, precision, sensitivity, specificity, PR‐AUC, balanced accuracy, and F1‐score. Confusion matrices were calculated for the held‐out test set. The 95% confidence intervals for performance metrics were estimated using 500 bootstrap resamples of the test‐set predictions. To evaluate feature‐selection robustness, bootstrap stability analysis was performed by repeatedly resampling the training set and re‐running the feature‐selection procedures. Given that this was a retrospective secondary analysis of a fixed public dataset, no prospective power calculation was performed before case accrual. The impact of the sample‐size limitation was therefore evaluated analytically through dimensionality reduction, held‐out test‐set evaluation, bootstrap confidence intervals, and bootstrap feature‐selection stability analysis.

## 3. Results and Discussion

### 3.1. KiTS23 Dataset and Radiomics Feature Reduction

The analysis included 396 cases after excluding multifocal cases to reduce lesion‐level label ambiguity and intrapatient lesion heterogeneity. The binary classification task was to distinguish ccRCC from non‐ccRCC renal tumors, including papillary RCC, chromophobe RCC, and oncocytoma.

Following the preprocessing and PyRadiomics extraction workflow, 386 radiomics features were initially extracted for each case. After reliability filtering, low‐variability feature removal, correlation pruning, and correlation‐distance‐based feature clustering, 73 candidate radiomics features were retained for model development. This reduction step was important because radiomics feature spaces are typically high‐dimensional and internally correlated. Reducing the feature set before model construction helped limit redundancy and improve the interpretability of subsequent classifier comparisons.

For the primary model, LASSO feature selection further reduced the candidate set to 6 features prior to class‐weighted Logistic Regression. The six retained features were original_gldm_DependenceEntropy, original_firstorder_Kurtosis, log‐sigma‐2‐0‐mm‐3D_firstorder_Kurtosis, log‐sigma‐2‐0‐mm‐3D_glcm_Imc2, log‐sigma‐3‐0‐mm‐3D_glszm_LowGrayLevelZoneEmphasis, and original_firstorder_Skewness. These descriptors included both first‐order intensity‐distribution features and texture features from original and LoG‐filtered images, suggesting that both intensity distribution and local heterogeneity contributed to discrimination between ccRCC and non‐ccRCC.

### 3.2. Classifier Model Evaluation

**FIGURE 3:**
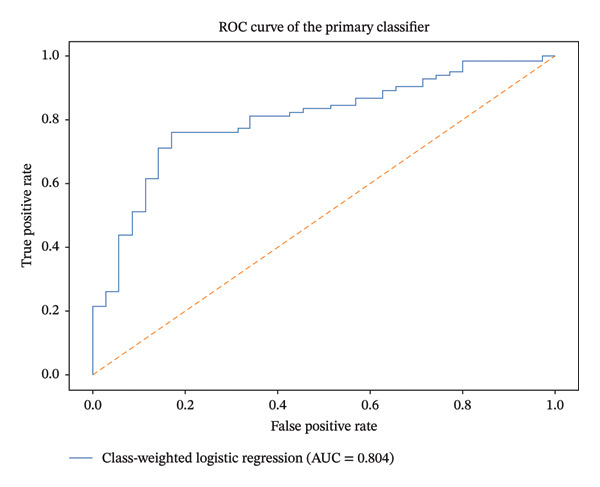
Receiver operating characteristic curve of the primary LASSO‐selected class‐weighted logistic regression model for differentiating ccRCC from non‐ccRCC renal tumors on the held‐out test set. The model achieved an ROC‐AUC of 80.4%.

The primary model was a class‐weighted Logistic Regression classifier using six LASSO‐selected radiomics features. As shown in Table 1, under the default probability threshold of 0.5, this model achieved an accuracy of 76.5%, an ROC‐AUC of 80.4%, a precision of 91.2%, a sensitivity of 73.8%, and a specificity of 82.9% on the held‐out test set. The corresponding ROC curve is shown in Figure 3. The relatively high precision and specificity indicate that the model had a favorable ability to reduce false‐positive classification of non‐ccRCC tumors as ccRCC. This operating characteristic is clinically relevant because false‐positive classification of ccRCC may bias interpretation towards a more aggressive malignant subtype.

**TABLE 1:** Evaluation scores of the primary LASSO‐selected class‐weighted logistic regression classifier under fixed and Youden‐index thresholds.

Metric (%)	LASSO + class‐weighted logistic regression	
Threshold	0.5	0.482
Accuracy	**76.5** (68.9–84.0)	74.8 (67.2–82.4)
ROC‐AUC	**80.4** (73.1–88.8)	**80.4** (73.1–88.8)
Precision	**91.2** (84.5–97.2)	85.5 (77.6–93.4)
Sensitivity	73.8 (64.0–82.7)	**77.4** (68.1–86.2)
Specificity	**82.9** (68.5–94.4)	68.6 (53.6–84.4)
PR‐AUC	**91.0** (85.9–95.8)	**91.0** (85.9–95.8)
Balanced accuracy	**78.3** (70.8–85.6)	73.0 (63.7–81.5)
F1‐score	**81.6** (75.0–87.8)	81.2 (74.7–87.7)

*Note:* Data are shown as point estimates with 95% confidence intervals. The higher of the two values was marked in bold.

The use of class weighting was important to the model’s operating profile, while the full model comparison is provided in Supporting Information (Table) S1. Among the LASSO‐based configurations, the unweighted Logistic Regression model achieved a comparable ROC‐AUC but showed a different sensitivity‐specificity profile, with higher sensitivity and lower specificity at the default threshold. By contrast, the class‐weighted Logistic Regression model shifted the decision profile toward improved specificity while yielding the highest ROC‐AUC among the evaluated configurations. This suggests that class weighting not only affected global discrimination but also improved the classifier’s clinical operating characteristics by reducing false‐positive predictions among non‐ccRCC tumors.

The comparison of feature‐selection strategies also supported the selection of LASSO for the primary model. As shown in Supporting Information (Table) S1, LASSO produced the highest ROC‐AUC while retaining the most compact feature set. Alternative strategies, including Logistic Regression‐based recursive feature elimination with cross‐validation, SVM‐based recursive feature elimination with cross‐validation, and mRMR, selected larger or different feature subsets but did not improve the primary discrimination performance. Some RFECV‐ and mRMR‐based configurations achieved competitive results in individual classifiers, but their performance was less consistent across threshold settings and classifier types. Therefore, the LASSO‐selected feature set was preferred because it yielded a compact, interpretable model with favorable specificity.

A Youden‐index thresholding strategy was also evaluated using cross‐validated out‐of‐fold predictions from the training set. For the LASSO‐selected class‐weighted Logistic Regression model, the Youden threshold was 0.482. As shown in Table 1, applying this threshold to the held‐out test set increased sensitivity but reduced specificity compared with the fixed threshold of 0.5. Since specificity was an important operating characteristic for reducing false‐positive ccRCC predictions among non‐ccRCC tumors, the fixed threshold of 0.5 was retained as the primary operating threshold. The ROC‐AUC remained unchanged across these two thresholding strategies because both thresholds were applied to the same predicted probabilities from the same classifier.

Across the evaluated model configurations, more complex classifiers did not clearly outperform class‐weighted Logistic Regression. Nonlinear and ensemble‐based models, including SVM‐RBF, Random Forest, Gradient Boosting, XGBoost, and LightGBM, did not demonstrate a clear edge over the LASSO‐selected Logistic Regression models. Some nonlinear models achieved higher sensitivity but often at the expense of specificity, indicating a tendency to classify a larger proportion of cases as ccRCC. Other configurations achieved higher specificity only at the cost of substantially reduced sensitivity. Taken together, these findings suggest that the selected radiomics feature space may encode a relatively linearly separable component of the ccRCC versus non‐ccRCC phenotype. This is consistent with the performance of Logistic Regression compared with more flexible nonlinear models and supports the use of a compact linear classifier in this modestly sized radiomics cohort.

Overall, the primary class‐weighted Logistic Regression model achieved moderate discrimination with favorable specificity and precision. This balance is important because the clinical usefulness of a ccRCC classifier depends not only on ROC‐AUC but also on its threshold‐dependent behavior. Although the results support the feasibility of radiomics‐based discrimination of ccRCC, the model has only been evaluated internally and requires validation in larger independent cohorts before clinical application.

### 3.3. Feature‐Selection Stability Analysis

**FIGURE 4:**
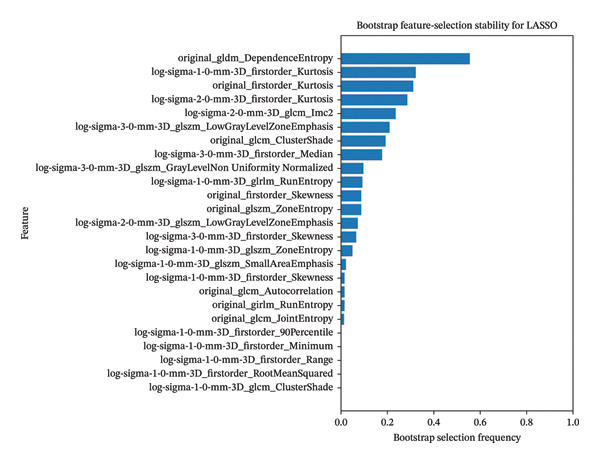
Bootstrap feature‐selection stability analysis for LASSO. Bars indicate the frequency with which each radiomics feature was selected across 200 bootstrap‐resampled training cohorts. The full ranking is provided in Supporting Table S2.

Bootstrap feature‐selection stability analysis was performed to evaluate whether the compact LASSO‐selected signature was repeatedly recovered across resampled training cohorts. As shown in Figure 4, several descriptors were repeatedly selected during bootstrap resampling. The full stability ranking is provided in Supporting Information (Table S2).

Among the six features retained in the primary model, original_gldm_DependenceEntropy showed the highest selection frequency, being retained in 111 of 200 bootstrap iterations. Other final‐model features also appeared among the more frequently selected descriptors, including original_firstorder_Kurtosis, log‐sigma‐2‐0‐mm‐3D_firstorder_Kurtosis, log‐sigma‐2‐0‐mm‐3D_glcm_Imc2, and log‐sigma‐3‐0‐mm‐3D_glszm_LowGrayLevelZoneEmphasis. Five of the six final LASSO‐selected features appeared among the six most frequently selected descriptors in the bootstrap analysis, supporting the internal consistency of the selected radiomics signature.

The stability analysis also indicates that individual feature‐level interpretations should be approached with caution. The selection frequencies were moderate rather than uniformly high, which is expected in radiomics datasets where many intensity and texture descriptors are correlated or partially interchangeable. For example, several first‐order kurtosis descriptors from different image filters were repeatedly selected, suggesting that related intensity‐distribution features may substitute for one another across resampled training sets. Therefore, the selected features should be interpreted primarily as components of a compact predictive radiomics signature rather than as individually definitive imaging biomarkers.

Together, the classifier evaluation and stability analysis suggest that the radiomics workflow identified a small, interpretable feature set capable of discriminating ccRCC from non‐ccRCC with favorable specificity. The findings remain exploratory and internally validated, but the use of dimensionality reduction, class‐weighted modeling, threshold evaluation, and bootstrap stability analysis strengthens the model’s methodological basis compared with an unregularized, high‐dimensional radiomics workflow.

### 3.4. Limitations

A further limitation is the relatively modest sample size and the absence of a prospective power or sample‐size calculation, given this was a retrospective secondary analysis of a fixed public dataset. This may affect the robustness of the reported performance estimates and limit the generalizability of the findings. Further validation in larger independent multicenter cohorts is therefore required.

## Author Contributions

Sourav Bhattacharjee had full access to the data used in this study and took responsibility for the data integrity and the accuracy of the analysis.

## Funding

This work was supported by the China Scholarship Council, 10.13039/501100004543.

## Disclosure

Sourav Bhattacharjee affirms that this manuscript is an honest, accurate, and transparent account of the study being reported; that no important aspects of the study have been omitted; and that any discrepancies from the study as planned (and, if relevant, registered) have been explained. All authors have read and approved the final version of the manuscript. The funding source was not involved in the study design; data collection, analysis, or interpretation; writing of the report; or decision to submit the article for publication.

## Conflicts of Interest

The authors declare no conflicts of interest.

## Supporting Information

Additional supporting information can be found online in the Supporting Information section.

## Supporting information


**Supporting Information 1** Supporting Information (Table) S1. Full model performance across feature‐selection strategies, classifiers, and threshold strategies. Data are shown as point estimates (95% confidence intervals).

**Supporting Information 2** Supporting Information (Table) S2. Bootstrap feature‐selection stability ranking for LASSO. The table reports the selection count, the number of completed bootstrap iterations, the selection frequency, and whether each feature was retained in the final primary model.

## Data Availability

The imaging data analyzed in this study are from the publicly available KiTS23 dataset. Access is provided by the organizers https://kits-challenge.org/kits23/, subject to their terms of use.
